# Chronic Inflammatory Demyelinating Polyradiculoneuropathy in Association With Concomitant Diseases: Identification and Management

**DOI:** 10.3389/fimmu.2022.890142

**Published:** 2022-07-04

**Authors:** Yan Chen, Xiangqi Tang

**Affiliations:** Department of Neurology, The Second Xiangya Hospital of Central South University, Changsha, China

**Keywords:** chronic inflammatory demyelinating polyradiculoneuropathy, diabetes mellitus, monoclonal gammopathy of undetermined significance, systemic lupus erythematosus, HIV, concomitant

## Abstract

Chronic inflammatory demyelinating polyradiculoneuropathy (CIDP) is a rare, heterogeneous, but treatable autoimmune-mediated peripheral neuropathy characterized by demyelination. CIDP can occur independently or simultaneously with a variety of diseases such as diabetes, monoclonal gammopathy of undetermined significance (MGUS), connective tissue disease, and HIV. It is important to identify CIDP and specific peripheral neuropathies caused by these diseases; this review aims to summarize the CIDP literatures related to diabetes, MGUS, SLE, and HIV, and to be helpful for the management of such patients.

## Introduction

Chronic inflammatory demyelinating polyradiculoneuropathy (CIDP) is a rare, heterogeneous, but treatable autoimmune-mediated peripheral neuropathy, resulting from a synergistic interaction of cellular and humoral immune responses. Although the etiology and exact pathogenesis remain elusive, it is well known that CIDP is characterized by a symmetric distal and proximal weakness, from distal to proximal extremities mostly, often with sensory disorder and the absence of or reduced tendon reflexes, and develops over at least 8 weeks (1–3). It was also known as chronic Guillain–Barre syndrome, first reported by Hinman and Magee in 1967 (4), and then named by Dyck in 1975 (5). In this article, the authors described the clinical, electrophysiological, and pathological features of CIDP, and for the first time defined CIDP as a separate disease entity.

Diabetes mellitus (DM) is a metabolic disorder characterized by chronic hyperglycemia; it can be associated with a number of peripheral nerve disorders, which include some inflammatory neuropathies (6). Monoclonal gammopathy is a group of disorders with excessive amounts of serum immunoglobulins produced by proliferation of a single clone of plasma cells. It can be divided into two parts: malignant monoclonal gammopathy and monoclonal gammopathy of undetermined significance (MGUS). Paraproteins may be accompanied by demyelinating or axonal neuropathies, and many demyelinating neuropathies are indistinguishable from CIDP. There is still no consensus on whether these two should be considered as the same disease or different diseases (7). Systemic lupus erythematosus (SLE) is a type of autoimmune inflammatory connective tissue disease that occurs more in young women and can involve multiple organs and multiple systems. The causes are still unclear and the clinical manifestations are diverse. A large number of studies have shown that it may be related to genetics, endocrine, infection, and immune abnormalities (8, 9). About half of SLE patients may be accompanied by different neuropsychiatric symptoms, called neuropsychiatric SLE. Deposition of autoantibodies at peripheral nerve components may trigger demyelination by macrophages of the peripheral nerves, which may be rare but cannot be ignored; CIDP should be taken into consideration.

There are many articles or reviews about the subtypes, antibody-related pathogenesis, diagnosis, and treatment of CIDP. Previous guidelines have highlighted CIDP in association with concomitant diseases that include diabetes, as well as other disorders such as IgG or IgA monoclonal gammopathy of unknown significance, IgM monoclonal gammopathy without anti-MAG (myelin associated glycoprotein) antibodies, connective tissue diseases, and human immunodeficiency virus (HIV) infection (10). Doneddu et al. (11) collected information on comorbidities from 393 CIDP patients fulfilling the European Federation of Neurological Societies/Peripheral Nerve Society (EFNS/PNS) criteria included in the Italian CIDP database; using a structured questionnaire, they found that diabetes (14%), MGUS (12%), and other immune disorders (16%) were significantly more frequent in patients with CIDP than expected in the general European population. The association of CIDP and these concomitant diseases may have both clinical and investigational implications. However, reviews about CIDP in concomitant diseases are limited; thus, our review aims to summarize these related literatures, and try to highlight the clinical importance of identifying and managing CIDP associated with such disorders.

## Overview of CIDP

### Chronic Inflammatory Demyelinating Polyradiculoneuropathy

The course of CIDP can be varied from gradually progressive, stepwise progressive, to relapsing–remitting (2). Both the cellular and humoral components of the immune system appear to be involved in the pathogenesis of CIDP and its variants (3). CIDP is a demyelinating neuropathy with myelin as the main target; the large nerve fibers containing the most myelin are predominantly affected while small nerve fibers with little or no myelin are largely unaffected. Its pathophysiology presents as segmental inflammatory demyelination that manifests as prolongation of distal motor latency, reduction of motor or sensory conduction, conduction block in some areas, temporal dispersion, prolongation of F-wave latency, absence of F-wave in electrodiagnostic criteria and as macrophage-mediated demyelination, onion-bulb formation (stacks of Schwann cell cytoplasmic processes), edema of endoneurium, and endoneurial inflammatory infiltration in nerve biopsy (2, 3, 10). Moreover, cerebrospinal fluid protein levels are generally elevated while cell counts are not elevated; this is also a supporting criterion for the diagnosis of CIDP (10).

There are currently many studies about the pathogenesis of CIDP; since the recognition of neurofascin-155 (NF-155), neurofascin-140 (NF-140), neurofascin-186 (NF-186), contactin-1 (CNTN-1), contactin-associated protein 1 (CASPR1), and other autoantibodies against the node of Ranvier or paranodal protein, we now have a better understanding of CIDP and promote the progress of diagnosis and treatment of CIDP (2, 3, 10, 12, 13). Interestingly, it was newly classified as an autoimmune nodopathy in the recent update of the European Academy of Neurology/Peripheral Nerve Society (EAN/PNS) CIDP guidelines, and not as a CIDP variant, because of its different clinical and pathological characteristics and response to CIDP treatment (10).

### Epidemiology of CIDP

The prevalence and incidence of CIDP vary in different countries and regions. Merel et al. conducted a systematic review and meta-analysis to estimate the prevalence and incidence of CIDP worldwide, and reported prevalence to range from 0.67 to 10.3 cases per 100,000 population and incidence to range from 0.15 to 1.6 cases per 100,000 person-years (14). Furthermore, they found a male predominance with increasing prevalence and incidence with age. The epidemiologic data of previous studies showed substantial variety, which may partly be due to the different diagnostic criteria (14, 15).

The cause of CIDP is still uncertain, although it is mainly attributed to an autoimmune reactivity against nerves. A previous preliminary study indicated that antecedent events, more specifically, antecedent infections or vaccinations, are more associated with an acute onset of CIDP and with cranial nerve involvement (16). Because of the limited epidemiologic data, more studies are needed to clarify the cause of CIDP and if the antecedent events are related to the onset of CIDP.

### Diagnosis Criteria of CIDP

The diagnosis of CIDP is challenging; in addition to the typical symmetric, sensorimotor CIDP phenotype, there are many CIDP variants such as sensory CIDP, motor CIDP, distal CIDP, multifocal CIDP, and focal CIDP (10). The clinical classification of CIDP phenotypes in different studies is diverse, and there is still no current consensus. Each phenotype has its own characteristics that results in many difficulties in diagnosing CIDP. The EFNS/PNS 2010 criteria are the most frequently used CIDP diagnosis criteria in clinical practice and research, which were revised recently, and now called “European Academy of Neurology/Peripheral Nerve Society guideline on diagnosis and treatment of CIDP”. The criteria are a combination of clinical, electrodiagnostic, and supportive criteria, and may provide higher diagnostic sensitivity (10, 17).

### Nerve Imaging Methods

Currently, there are new nerve imaging methods available for auxiliary diagnosis of CIDP under study, particularly of suspected CIDP, including nerve magnetic resonance imaging (MRI) and nerve ultrasound (18–21). They have their own unique advantages or drawbacks. Nerve MRI can identify gadolinium enhancement and/or hypertrophy of nerve roots or plexuses. Shah et al. (22) compared the MRI measures of the lumbosacral and sciatic nerves in 10 CIDP patients and 10 healthy controls, and demonstrated significant hypertrophy of the lumbosacral nerve roots and sciatic nerves in CIDP patients compared to healthy controls, which was consistent with the findings of Lichtenstein and colleagues (23). Diffusion tensor imaging (DTI) of the peripheral nerve analysis appears to provide some diagnostic value in CIDP in some studies (23, 24), but more research is needed. However, nerve MRI is expensive, is time-consuming, and requires a specialized radiologist to read the abnormalities of pictures compared to nerve ultrasound. In some previous studies, nerve ultrasound can accurately discriminate segmental demyelinating and disintegration, and fracture and atrophy of correlative muscle fasciculus, and provide some clues for the judgment of disease severity, the selection of the treatment regimen, and the evaluation of prognosis to some extent. Nerve ultrasound can observe an increase in peripheral nerve size over time especially in demyelinated nerve segments and correlates with clinical and electrodiagnostic changes (25, 26). The latest EAN/PNS CIDP criteria have acknowledged the application of MRI and ultrasound in possible adult CIDP patients, while systematic studies in pediatric patients are lacking.

### Management of CIDP

The current first-line treatment of CIDP includes intravenous immunoglobulin (IVIG), corticosteroids, and plasma exchange (PE) (1, 2, 27), and now, subcutaneous immunoglobulin (SCIG) has been gradually used for the maintenance treatment of CIDP patients who respond well to IVIG due to its similar efficacy and convenience, reducing infusion-related side effects (28, 29). However, more than 20% of patients do not respond to standard therapies, other biological agents, or immunosuppressive agents such as azathioprine, cyclophosphamide, ciclosporin, mycophenolate mofetil, and rituximab, which can be used as a secondary therapy when the first-line treatment was not effective or tolerated (1, 2, 10, 12). There are still insufficient randomized controlled studies about azathioprine, interferon β-1a, and methotrexate to provide strong enough support for clinical application (10, 30, 31). The latest EAN/PNS CIDP guidelines suggest that autoimmune nodopathy patients with IgG4 autoantibodies show better response to rituximab than IVIG or corticosteroids. It is worth mentioning here that autologous hematopoietic stem cell transplantation (AHSCT) has been a promising treatment approach for autoimmune diseases that included CIDP (32, 33), but still lack sufficient large studies.

## CIDP in Diabetes Mellitus

Diabetes mellitus (DM) is a group of metabolic diseases caused by genetic and environmental factors with chronic hyperglycemia as the main clinical manifestation. Diabetic patients with poor control of blood glucose commonly occur with a wide variety of peripheral neuropathies (6, 34), such as diabetic polyneuropathy (DPN), which is the most common diabetes-related peripheral neuropathy, diabetic radiculoplexus neuropathies (lumbosacral, thoracic, and cervical), acute painful neuropathy, insulin neuritis, diabetic autonomic neuropathy, and CIDP with diabetes (DM-CIDP).

CIDP is the most common treatable inflammatory neuropathy in diabetic patients, and is often compared with other diabetic peripheral neuropathies.

The possible relationship between DM and CIDP, as well as its clinical significance, has been a controversial topic. This review will summarize previous literature and hopes to be helpful for the clinical identification and management of DM-CIDP.

### The Possibility of Co-Association Between CIDP and DM

From previous preliminary studies, we can conclude that CIDP and diabetes may co-occur. The prevalence of CIDP tends to be higher in diabetic patients than in non-diabetic patients, especially in those of older age (35–39). From a study of Sharma et al. (36) in a population of patients in their electrophysiology laboratory, we found that the prevalence of CIDP in the DM population was 11 times higher than in the non-DM population; these data are questionable due to the limited research. Moreover, in a descriptive study by Bril et al. (35), the authors included CIDP patients of the 2009–2013 PharMetrics Plus™ Database and found that the prevalence of CIDP with DM was 9-fold higher than without DM (0.054% versus 0.006%), which is more apparent in patients over age 50 years, resulting from the average age of morbidity of type 2 diabetes. The conclusion is consistent with the findings of Gorson and colleagues (37). A European multicenter study by Rajabally et al. (40) retrospectively analyzed two European cohorts from Serbia and Birmingham, UK, with a total of 257 patients with definite or probable CIDP. Compared with the general population, CIDP patients had a twofold increased relative risk of diabetes, presented older, and were more frequently in typical form. DM may be a possible risk factor of CIDP.

However, some studies have found many contradictory results. An epidemiological study on CIDP in two Italian regions was performed by Chio et al. (41); they found that, among 155 patients with CIDP, 14 patients were affected by either type 1 or type 2 diabetes (9%). The expected number of individuals with associated DM was 13.03, corresponding to a standardized morbidity ratio of 1.07 [95% confidence interval (CI), 0.58–1.80]. It can be seen that the two values are very close, indicating that the prevalence of DM in patients with CIDP is close to that of the general population. CIDP and DM do not show a significant correlation of pathogenesis. Laughlin et al. (42) estimated the incidence of CIDP in Olmsted County, Minnesota from 1982 to 2001 and its prevalence on January 1, 2000. A total of 1,581 patients were reviewed, of whom 23 patients were identified as having CIDP, and only 1 of 23 CIDP patients had DM; the prevalence of DM-CIDP was significantly lower than that of 115 sex- and age- matched controls (4% versus 12%). Thus, they hypothesized that DM is unlikely to be a major risk covariate for CIDP, but a weak effect cannot be excluded. The perceived association between CIDP and DM may be due to misclassification of other diabetic neuropathies or excessive emphasis on electrophysiological diagnostic criteria (6, 42). Therefore, it is still unclear if this represents a causal relationship or a coincidental phenomenon.

### The Identification of DM-CIDP

Some patients with diabetic peripheral neuropathy may have CIDP-like electrophysiological manifestation such as demyelination. There is still no consensus on whether these patients have DM-CIDP or another demyelinating peripheral neuropathy caused by diabetes (37, 39). Dunnigan et al. (43) compared the clinical and electrophysiological features of 67 patients with DM-CIDP and 56 patients with “demyelinating” diabetic sensorimotor polyneuropathy (D-DSP), and they found that patients with DM-CIDP were older, had shorter duration of diabetes, had better control of blood glucose, but had more severe neuropathy. Overall, since CIDP is a disease that can be treated by immunotherapy (1–3, 10), it is very important to distinguish CIDP from other forms of peripheral neuropathies in diabetic patients (44, 45).

As mentioned above, typical idiopathic CIDP is mainly characterized by varying degrees of limb weakness, most of which are symmetrical, with both proximal and distal involvement, accompanied by paresthesia, without or with reduced tendon reflexes. In a study comparing 14 patients with DM-CIDP (10 male patients and 4 female patients) and 60 patients with idiopathic CIDP (37), Gorson et al. found that the symptoms and neurologic signs of DM-CIDP patients are similar to those of idiopathic CIDP patients, but with more severe imbalances and axonal loss. Patients with DM-CIDP showed similar inflammatory demyelination in electrophysiological studies that manifests as slowed motor conduction velocities, prolongation of distal latency, and prolonged or absent F wave, but with a more obvious decrease in CMAP and SNAP amplitudes, possibly as a result of more diabetic axonal damages (37, 39, 41, 43, 46, 47). Identifying CIDP in DM patients can be very difficult because many different types of neuropathy occur in association with DM. The diagnosis of CIDP in diabetic patients is often delayed compared to idiopathic CIDP.

Diabetic peripheral neuropathy is a chronic, progressive, distal symmetric axonal polyneuropathy affecting small fibers only or both small and large fibers. Insidious onset and characteristic features such as progressive exacerbation of numbness and paresthesia of distal extremities are mostly manifested as stocking-like or glove-like distribution, as well as neuropathic pain and autonomic dysfunction (e.g., gastrointestinal and cardiovascular) (6). Its pathogenesis is multifactorial and remains undefined (34), mostly thought to be caused by the metabolism and microvascular effects of chronic hyperglycemia. Immune and inflammatory factors may also play a role in diabetic peripheral neuropathy (6). The characteristics of diabetic peripheral neuropathy coincide with length-dependent axonal peripheral neuropathy and reduced sensory nerve action potential amplitude, generally without CIDP features such as motor nerve conduction block and abnormal temporal dispersion. When patients complained of painless, progressive, distal, and proximal limb weakness, and subacute deterioration, as well as a shorter course of diabetes, the diagnosis of CIDP should be considered (45, 48). The common autoantigens in peri-islet and peripheral nerve Schwann cells could serve as a possible pathogenic link between DM and CIDP (49). However, the identification of DM-CIDP in the clinic is still very challenging because of the lack of clear biomarkers.

Stewart et al. (39) collected data from 7 patients with DM who met clinical and electrophysiological diagnostic criteria for CIDP; at the same time, nerve conduction studies and nerve biopsies were performed in all 7 patients. They all showed varying degrees of reduction of motor conduction velocity or conduction block, prolonged distal motor latency, and absent or delayed F wave. Sural nerve biopsy showed a variety of abnormalities, but none of which distinguished CIDP from diabetic neuropathy clearly. Jann et al. (45) suggested that immunoreactivity for matrix metalloproteinase-9 (MMP-9) on sural nerve biopsies may help in identifying patients with DM-CIDP. Sural nerve biopsies in DM-CIDP patients showed more MMP-9 positive in endoneurial vessels than diabetic neuropathy patients, as well as in the epineurium. Latov (44) found that some specific mRNA transcripts in the nerve and skin of DM-CIDP patients may contribute to the differential diagnosis of diabetic peripheral neuropathy. Fleischer et al. (50) compared corneal confocal microscopy (CCM) results between CIDP, DM-CIDP, and diabetic neuropathy patients in a multicenter case–control study, and found that dendritic cell density in proximity to corneal nerve fibers was significantly higher in participants with CIDP with and without diabetes compared to those with diabetic neuropathy. Hence, CCM may help to differentiate inflammatory from non-inflammatory diabetic neuropathy. Lotan and colleagues (51) proposed a diagnostic tool to help clinicians to identify patients with DM-CIDP by combining several different clinical, electrophysiological, and laboratory parameters, and applied it in three groups of patients (those with DM, CIDP, and DM-CIDP); at last, its validity was determined. Due to the relatively limited data, further large studies are needed, but this undoubtedly brings hope to the majority of clinicians for the identification of CIDP. Currently, there are no specific diagnostic criteria for DM-CIDP patients; identification of DM-CIDP is based on clinical, electrophysiological, laboratory, and nerve imaging information, combined with CIDP diagnostic criteria (10, 17, 52). Rajabally et al. (52) believed that a treatment trial can be performed when there is a high suspicion of CIDP, especially with disabling neuropathy. Patients with DM-CIDP usually respond well to immunotherapy of CIDP and clinically improve.

### The Management of DM-CIDP

Although there is still no clear correlation between CIDP and DM, the coexistence of both diseases increases the challenge of treatment (34). Glycemia control and symptomatic treatment are the main treatment for patients with diabetic peripheral neuropathy (34), while the first-line therapy of DM-CIDP patients is similar to idiopathic CIDP patients, including IVIG, corticosteroids, and plasma exchange (PE); in some refractory cases, immunosuppressive agents may be considered (46, 52).

Treatment for DM-CIDP should be individually administered, and the most appropriate treatment should be selected according to the patient’s specific manifestations, severity, and other comorbidities. Especially for those elderly patients or patients with severe cardiac and renal insufficiency, vital signs and condition changes should be closely observed, water and electrolyte balance should be controlled, and the treatment plan should be adjusted in time to reduce the occurrence of complications. IVIG is often chosen by clinicians to treat DM-CIDP patients, especially in some severe cases (52). In a study by Haq et al. (47), 6 patients with DM-CIDP received immunomodulatory therapy, and all showed favorable therapeutic response. Jann and coworkers (45) also found that 10 patients with DM-CIDP responded well to IVIG treatment under strict control of glycemia, which is consistent with another prospective study (53). Sharma et al. (36) designed an uncontrolled study to evaluate the responsiveness to IVIG treatment of DM-CIDP patients. A total of 26 patients with type 2 diabetes met the CIDP diagnostic criteria and received IVIG 0.4 g/kg/day for 5 days. After 4 weeks, 21 patients (80.8%) had a significant clinical improvement measured by the neuropathy impairment score compared with baseline (mean [standard deviation], 61.5 [26.0] versus 33 [29.6], *p* < 0.001). However, further controlled trials are still needed to confirm this. Furthermore, the efficacy of IVIG in DM-CIDP patients is in keeping with other studies (54, 55). Samantha et al. (46) compared the efficacy of IVIG in 134 patients with CIDP (67 of whom had DM) and found no significant difference between the two populations. Interestingly, only one report described the inferiority of IVIG. Pedersen and coworkers (56) reported that a 54-year-old type 2 diabetic patient with CIDP deteriorated after two courses of IVIG treatment. The patient switched to corticosteroid therapy for 1 month and had improved significantly. Therefore, IVIG shows a certain efficacy in the treatment of DM-CIDP; nevertheless, the treatment regimen should be individualized according to the actual situation. Abraham et al. (57, 58) considered that the responsiveness to IVIG treatment of DM-CIDP patients was attributed to a high degree of demyelination. Whether IVIG treatment is necessarily superior to other treatment options remains a big issue due to its drawbacks such as hospitalization, high cost, and possible adverse effects, such as renal failure, flu-like symptoms, headache, and embolism events; larger comparative studies are required to further confirm this (59, 60).

As far as we know, there are insufficient studies on the use of corticosteroids in patients with DM-CIDP. Because of the impact of corticosteroids to glycemic control, DM-CIDP patients should be carefully monitored when treated with this therapy. Corticosteroid therapy should be avoided in the presence of very poor control of blood glucose, infection, hyperadrenocorticism, peptic ulcer, etc. According to long-term clinical experience, there are many schemes with different dosages or drugs for steroid therapy, for instance, oral dexamethasone pulse treatment, daily oral of high-dose prednisolone, or intravenous methylprednisolone (52, 59), but maintenance treatments that last for years are often required. Patients need to be monitored regularly in case of osteoporosis, full moon face, obesity, etc. Plasma exchange (PE) is difficult for DM-CIDP due to its venous access, and because its efficacy is short term, it necessitates repeated treatment, and should not be considered as a routine treatment option (52). Münch et al. (61) reported a poor efficacy of IVIG in a 57-year-old patient with DM-CIDP who successfully benefited from rituximab treatment; she maintained disease stability during the 10 months of follow-up, suggesting that rituximab may be a new alternative to DM-CIDP therapy, and more controlled trials are required.

## CIDP in Monoclonal Gammopathy of Undetermined Significance

The patients with monoclonal gammopathy have a large number of monoclonal immunoglobulins (M protein) in the serum, including various anti-myelin antibodies. The interaction of these antibodies with specific antigenic targets on peripheral nerves or deposition of immunoglobulins will lead to neuropathy, also known as paraproteinemic neuropathy (62). The association of peripheral neuropathy with monoclonal gammopathy has been previously demonstrated; however, its clinical and pathogenetic relevance as well as therapeutic implications are still far from having been established (63). Neurologists are more concerned with neuropathy in patients with monoclonal gammopathy of undetermined significance (MGUS) than with malignant paraproteinemia (64). It is still debated whether neuropathies associated with MGUS should be considered entity diseases or included among CIDP or chronic idiopathic axonal polyneuropathy (63).

Byun et al. (65) retrospectively analyzed the clinical data of 18 MGUS patients, 15 POEMS syndrome patients, and 34 CIDP patients between January 2005 and December 2016, and they found that patients with plasma cell disorder-related neuropathy seemed to be older and more sensory oriented compared to CIDP patients; moreover, patients with POEMS syndrome showed significantly higher platelet counts.

Some patients with atypical CIDP may coexist with MGUS (CIDP-MGUS) (1, 66–68). Compared with idiopathic CIDP, CIDP-MGUS is more common in older men, and there were no significant differences in motor and sensory nerve conduction studies (68). Simmons and colleagues (67) performed a long-term follow-up (at least 24 months) of 69 patients with idiopathic CIDP and 25 patients with CIDP-MGUS. Most of the CIDP-MGUS patients showed a slower progressive course with less functional impairment than idiopathic CIDP, and more sensory involvement. The strength and functional scores after IVIG treatment were poorer in CIDP-MGUS than idiopathic CIDP. A study of Gorson et al. (66) showed that the muscle weakness of CIDP-MGUS patients were relatively milder, with greater imbalance, ataxia, and vibration loss, and with responsiveness to immunotherapy similar to idiopathic CIDP; however, functional improvement was greatest after PE treatment. A study (69) aiming to investigate the use of peripheral nerve ultrasound in the differentiation of paraproteinemic neuropathies found that patients with CIDP had the most marked nerve enlargement; thus, we hypothesize that peripheral nerve ultrasound may be an additional approach to identify CIDP-MGUS; further studies are needed to confirm its validity.

### Paraproteinemic Demyelinating Neuropathies

The clinical and electrophysiological characteristics of CIDP-MGUS are very similar to some paraproteinemic demyelinating neuropathies (PDNs). They may have the same pathophysiological mechanisms and are difficult to identify (7). Notermans and coworkers proposed a diagnostic criteria for MGUS-related demyelinating neuropathy (70). CIDP-MGUS patients were characterized by a chronic progressive course, more involved sensory symptoms and signs, and no cranial nerve involvement.

MGUS associated with demyelinating neuropathy is the most common PDN. It is mainly divided into two types according to the type of paraprotein: PDN associated with IgM MGUS and PDN associated with IgG/IgA MGUS (7).

IgM PDN is a chronic progressive disease with clinical manifestations of distal, symmetrical sensory involvement and ataxia, without or with minimal motor involvement. In addition, about 50% patients have anti-myelin-associated glycoprotein (anti-MAG) IgM antibodies in the serum or biopsies, and may have a different pathogenesis to CIDP (71). The following conditions may be helpful in the differential diagnosis of IgM PDN and CIDP (7): (1) uniform symmetrical reduction of conduction velocities (more severe sensory than motor involvement); (2) distal motor latency (DML) is prolonged disproportionately; (3) absence of sural potential; and (4) partial conduction block and marked distal composite muscle action potential (CMAP) dispersion are very rare. These patients are difficult to treat with poor response to immunosuppressive therapy. According to individual conditions and whether there are comorbidities, the pros and cons must be weighed before choosing the appropriate management. A proportion of patients with IgM MGUS may exhibit CIDP-like features; they are considered to be a class of atypical CIDP variants—distal, acquired demyelinating symmetric neuropathy (DADS) because of their manifestations of distal, symmetric, sensory involvement, ataxia, and tremor, whose responsiveness is similar to typical CIDP (72, 73).

IgG/IgA PDN is a more progressive disease than IgM PDN, often with a relapsing–remitting course. It is characterized by the weakness of proximal and distal muscles of the extremities, the simultaneous involvement of motor and sensory nerves, the absence of specific antibodies, and difficulty in distinguishing from CIDP in terms of clinical and electrophysiological aspects. The treatment principle is the same as that of CIDP (7, 74). The good response of patients with IgG PDN to IVIG may be related to proximal muscle weakness, the short course of the disease, and electrophysiological demyelination (75).

### The Management of CIDP-MGUS

The responsiveness of CIDP-MGUS to immunotherapy is different depending on the types of monoclonal gammopathy. The use of steroids, immunosuppressive agents, PE, or IVIG alone or combined can be considered for the treatment of CIDP-MGUS (62, 76). The efficacy of IVIG in patients with IgG/IgA MGUS is better than that in patients with IgM MGUS. Patients with axonal damages have a poorer response to immunotherapy. Patients with neuropathy with anti-MAG antibodies and a small proportion of patients with IgA MGUS neuropathy have a worse response to immunomodulatory therapy and may be associated with severe side effects; thus, identification is very crucial (74).

Jann et al. (53) compared the treatment response of IVIG in 16 patients with DM-CIDP, 7 patients with CIDP-MGUS, and 8 patients with idiopathic CIDP. Patients with CIDP-MGUS were found to have a more dangerous onset and less severe but longer course of disease and relapse more frequently. Hayashi et al. (77) reported a 71-year-old female patient with CIDP and IgA-λ MGUS in 2016 who developed quadriplegia, respiratory failure, and rapid fluctuations in blood pressure. After a large dose of corticosteroids and IVIG treatment, the patient’s symptoms improved, but quickly relapsed, and showed immunoglobulin IgA-λ and IgM-λ double positive in the serum. This was followed by treatment with PE, dexamethasone, methotrexate, etc., after which the symptoms of the patient did not improve significantly, and various anti-ganglioside IgM antibodies (GD3 +++, GT1a ++++, GT1b ++, GQ1b +++, and GD1b +++) gradually appeared. Surprisingly, the serum IgA-λ monoclonal protein gradually transformed to the strongly positive IgM-λ M protein. After 12 months of IVIG treatment, the patient’s neurological symptoms gradually improved. This is the first reported case of CIDP with MGUS showing an alternating immunoglobulin class.

Posa et al. (78) reported a case of rituximab in CIDP-MGUS patients and found that it had a favorable effect on the disease course, clinically and in nerve conduction studies. Colucci et al. (79) found that autologous hematopoietic stem cells had a favorable effect on the control of neurological symptoms in patients with CIDP-MGUS. Of course, further evaluation in multicenter prospective studies is needed.

## CIDP in Systemic Lupus Erythematosus

CIDP is rarely reported in connective tissue diseases; the most common concomitant disease is SLE. About 10%–20% of patients with SLE can be associated with peripheral neuropathy (9), and more manifest as having symmetric sensorimotor polyneuropathy (80). Sensory symptoms can be expressed as numbness, tingling, or pain, and motor symptoms can be manifested as weakness of regional muscles and affected nerves, such as wrist or foot drop (81). Nerve biopsy can be characterized with axonal degeneration or depletion, which can be accompanied by non-specific microvascular changes or perivascular inflammation, and some patients may also have demyelinating changes (9). To the best of our knowledge, previous reports of peripheral nervous system involvement in SLE are rare. According to the American College of Rheumatology (ACR) case definitions (82), common peripheral neuropathies of SLE include the following 7 types: acute inflammatory demyelinating polyradiculoneuropathy, autonomic disorder, mononeuropathy, myasthenia gravis, cranial neuropathy, plexopathy, and polyneuropathy. Other rare peripheral neuropathies, such as CIDP and small fiber neuropathy, are not well characterized in SLE and are not included in the ACR nomenclature (83).

The pathogenesis of SLE-associated peripheral nervous system diseases may be related to ischemia, immune complex deposition, antibody-mediated reactions, demyelination or abnormal metabolism, or autoimmune response-like vasculitis caused by connective tissue diseases (8, 81). Neuropathies are more common in antibody-positive patients. Some researchers (84) suggest that GM-1 and GM-3 autoantibodies may play an important role in the pathogenesis of CIDP in SLE patients. However, due to insufficient evidence, the pathogenesis correlation between SLE-related autoantibodies and peripheral neuropathy in SLE patients is still unclear (80). Earlier identification of SLE with CIDP can help early diagnosis and treatment of patients and achieve clinical improvement.

CIDP can appear before, after, or at the same time as SLE, and the causes of the disease are varied and are not clear. In addition to the typical clinical manifestations of CIDP, SLE patients with CIDP may also present recurrent episodes of Guillain–Barré syndrome-like symptoms (85, 86), which may range from weeks to months of the disease course. At this point, distinguishing the two is extremely difficult, and nerve conduction studies must be performed, which is especially important for identifying the type of neuropathy. In most cases, both upper and lower limbs are involved at the same time; electrophysiological and nerve biopsy shows typical demyelinating manifestations. Apart from common skin or musculoskeletal symptoms in SLE patients, half may be accompanied by involvement of visceral organs (87). Julio et al. reviewed the literature on CIDP associated with SLE prior to December 2019 and identified a total of 10 articles, including 16 patients. Besides typical CIDP clinical manifestations, there was a predominance of women with SLE disease activity, especially nephritis and hematological involvement (88).

A variety of immunomodulatory therapies can be selected for the treatment of SLE with CIDP. A study from Abraham et al. (89) reported that a 40-year-old female patient with SLE and CIDP showed bilateral progressive, ascending sensorimotor neuropathy, accompanied by ANA, anti-Sm, anti-RNP, anti-SSA, and anti-dsDNA antibodies positive in the serum; her neurological symptoms improved significantly after IVIG and high-dose steroid therapy. In a study of 6 patients with SLE and CIDP from Vina (87), 3 patients achieved substantial clinical response to IVIG, and their CIDP characteristics, involvement of critical internal organs, and multiple autoantibodies associated with SLE may predict a good response to IVIG. Aside from IVIG and steroid therapy, other immunotherapies are also taken into consideration. Zoilo et al. (90) described a male patient with adolescent SLE and CIDP who was diagnosed with SLE at the age of 13 and developed weakness of upper extremities, which then progressed to all extremities, and tendon reflexes disappeared without central nervous system involvement. The boy was diagnosed as having demyelinating polyneuropathy in the end; serum antibodies against ANA, DNAdc, beta2 glycoprotein, cardiolipin, and ANCA-C/X were positive. After receiving IVIG, cyclophosphamide, and daily steroid and azathioprine treatment for 2 weeks, significant clinical improvement was obtained and he was discharged. Jasmin et al. (91) reported on a 26-year-old male patient with SLE and CIDP who received IVIG treatment but proved to be ineffective and the disease progressed, while patients improved after being treated with a combination of steroids and oral cyclophosphamide. In addition, monoclonal antibodies may also be an option for the treatment of this disease. Sanz et al. (92) found that rituximab was used to treat a 28-year-old female patient with SLE and CIDP and showed a good outcome.

However, there are a few studies on the treatment options for CIDP with SLE, and only a few case reports; thus, more multicenter cohort studies are needed. When CIDP patients with SLE show disease activity, corticosteroids or immunosuppressive drugs should be actively used, and IVIG can be considered when they are not effective (88). In the absence of SLE disease activity, standard CIDP treatment should be prescribed.

## CIDP in HIV

Various peripheral neuropathies can be caused by HIV infection and peripheral neuropathy is probably the most common neurological complication associated with HIV infection (93). Clinical presentations of HIV-related peripheral neuropathies include the following: distal symmetrical polyneuropathy; acute inflammatory demyelinating polyradiculoneuropathy (AIDP) and CIDP; mononeuropathies, mononeuropathies multiplex, and cranial neuropathies; autonomic neuropathy; lumbosacral polyradiculomyelopathy; and amyotrophic lateral sclerosis-like motor neuropathy.

The clinical and electrophysiological manifestations of HIV-positive CIDP patients were not significantly different from those of HIV-negative patients, showing a similar degree of demyelination, and there is a predominance of T lymphocyte or macrophage infiltration with CD8-positive cytotoxic/suppressor cells. CIDP may occur during late infection, often with a CD4 count of less than 50 cells/mm^3^.

In a prospective case series analysis of CIDP patients from Johannesburg, South Africa, Mochan et al. (94) found that 10 of 23 CIDP patients (43%) were HIV-positive and had similar clinical and electrophysiological characteristics compared with HIV-negative patients. They also found that HIV-positive status was associated with a progressive disease course and was significantly associated with CSF lymphocytic pleocytosis (*p* = 0.007).

Moodley et al. (95) performed a retrospective analysis in 2 neuromuscular units in Kwa-Zulu Natal between 2003 and 2015, and they found that 39 of 84 CIDP patients were HIV-infected; the majority of these patients were younger, were female, and presented with a monophasic progressive disease. Corticosteroid therapy was effective in 86% of patients, and 76% were in remission within 6–12 months without further treatment. Among HIV-negative patients, the majority were older, were male, and had a relapsing–remitting course. Twenty-seven percent of patients had a favorable response to corticosteroids, 95% required combination therapy, and 33% were not in remission by 18 months on therapy.

## CIDP in Other Comorbidities

To the best of our knowledge, there are very limited reports on CIDP complicated by polyneuropathy, organomegaly, endocrinopathy, monoclonal gammopathy, skin change (POEMS) syndrome, multiple myeloma (96), amyloidosis (97), malignant paraproteinemia, and other diseases. Part of the literature summarized the identification of CIDP with these diseases (2, 98–100). When CIDP-like neuropathies appear, certain characteristic features such as organomegaly, endocrine changes, amyloid deposition, severe axonal damage, tumor infiltration, and pain can help in the differential diagnosis, supplemented by auxiliary examinations. In addition, serum vascular endothelial growth factor (VEGF) testing in patients with acquired demyelinating neuropathy may facilitate early identification of POEMS (101).

## Summary

CIDP can coexist with a variety of diseases such as diabetes, MGUS, SLE, and other connective tissue diseases such as Sjogren’s syndrome, active hepatitis, HIV, thyroid disease, inflammatory bowel disease, kidney disease, and others. Clinicians should pay attention to the difference between CIDP in concomitant diseases and idiopathic CIDP or other similar neuropathy; early identification can assist clinicians in their therapeutic decision, so that patients can reduce their pain and get the maximum benefit. **Table 1** summarizes the manifestations of CIDP in different comorbidities and the treatment options available.

**Table 1:** The identification and management of CIDP associated with concomitant diseases.

Concomitant diseases	Clinical presentations and auxiliary examinations	Treatment
CIDP in DM (37, 43, 45, 50)	High blood glucose or elevated HbA1c; Typical CIDP features; Older patients; Better glycemic control; Shorter duration of diabetes; More serious imbalance; More severe axonal loss; Greater degrees of demyelination of peripheral nerves; More severe neuropathy phenotype (greater weakness, more abnormal reflexes, higher TCNS scores, more abnormal NCS); Epineurial perivascular T-cell infiltrates; Possible increased endoneurial MMP-9 immunoreactivity in sural nerve biopsy; CCM might be useful in some cases	IVIG (first-line therapy); PE; Cyclophosphamide; Rituximab; Corticosteroids (use with caution)
CIDP in MGUS (66, 67, 69, 78, 79)	Paraproteinemia; Suspicious CIDP clinical and demyelination manifestations; Elder men; Developed more slowly; Sensory impairment more obviously; Less severe weakness; Greater imbalance, ataxia, and vibration loss; Anti-MAG antibody testing may be useful; Peripheral nerve ultrasound may be an additional approach	IVIG; PE; Rituximab; Autologous hematopoietic stem cell transplantation may be a treatment option
CIDP in SLE (85, 86, 88–90)	Malar rash; Nephritis; Hematological involvement; Positive autoantibodies (e.g., ANA, dsDNA, anti-Sm); Typical clinical manifestations of CIDP; Recurrent episodes of Guillain-Barré syndrome-like symptoms in some cases; Electrophysiological and nerve biopsy show typical demyelinating manifestations; Albumin-cytological dissociation of CSF	IVIG; Corticosteroids; Cyclophosphamide; Azathioprine; Rituximab
CIDP in HIV (94, 95)	Younger; More female; Monophasic progressive course; CSF lymphocytic pleocytosis	Corticosteroids; IVIG; PE

There must be co-association between diabetes and CIDP to some extent. The occurrence of CIDP in DM patients may be related to antibodies against the common autoantigens of the peri-islet Schwann cells and peripheral nerve Schwann cells. It is particularly important to identify patients with DM-CIDP in DM with peripheral neuropathy, which is challenging despite the combined clinical, electrophysiological, pathological, and nerve imaging. The preferred treatment for patients with DM-CIDP is still immunotherapy, such as IVIG, steroids, PE, and immunosuppressive agents. Moreover, monoclonal antibodies like rituximab may have an active effect on DM-CIDP patients, but still need to be further determined. Furthermore, patients with DM-CIDP may have poorer response to IVIG resulting from more axonal injuries.

Paraproteinemia patients have a variety of antibodies on the serum against the myelin sheath or axonal membrane of nerves; thus, there is a possibility of CIDP-MGUS, which is difficult to distinguish from other demyelinating peripheral neuropathies. Attention should be paid to the identification of different types of immunoglobulin-related demyelinating peripheral neuropathies. At present, the primary treatment remains immunotherapy of CIDP-MGUS, and patients with IgG/IgA have a better response to IVIG than patients with IgM.

Reports about the connective tissue disease SLE coexisting with CIDP are very rare, and such occurrences are more frequent in antibody-positive SLE patients, which could be associated with autoantibodies or abnormal immunoreactions. Immunotherapy is the primary mode of treatment (IVIG is preferred) of SLE-CIDP patients. The typical CIDP features and SLE-related visceral involvement or autoantibodies may predict a good therapeutic response.

Peripheral neuropathy is probably the most common neurological complication associated with HIV infection, and CIDP mostly occurs in the later stages of HIV infection. Currently, limited studies have found that compared with HIV-negative patients, CIDP-HIV patients have no significant difference in clinical and electrophysiological manifestations, which can be manifested as monophasic progressive disease and CSF lymphocytic pleocytosis. The preferred treatment remains corticosteroid therapy, and further studies are needed.

## Conclusion

The current evidence-based data on CIDP with concurrent illness are still very limited, especially in the differential diagnosis and treatment of some rare concomitant diseases; more large studies are thus still needed.

## Author Contributions

YC contributed to manuscript preparation and wrote the manuscript. XT helped perform the analysis with constructive discussions. All authors contributed to the article and approved the submitted version.

## Conflict of Interest

The authors declare that the research was conducted in the absence of any commercial or financial relationships that could be construed as a potential conflict of interest.

## Publisher’s Note

All claims expressed in this article are solely those of the authors and do not necessarily represent those of their affiliated organizations, or those of the publisher, the editors and the reviewers. Any product that may be evaluated in this article, or claim that may be made by its manufacturer, is not guaranteed or endorsed by the publisher.

## Abbreviations

CIDP, chronic inflammatory demyelinating polyradiculoneuropathy; DM, diabetes mellitus; MGUS, monoclonal gammopathy of undetermined significance; SLE, systemic lupus erythematosus; DADS, distal acquired demyelinating symmetric neuropathy; EAN/PNS, European Academy of Neurology/Peripheral Nerve Society; EFNS/PNS, European Federation of Neurological Societies/Peripheral Nerve Society; MRI, magnetic resonance imaging; IVIG, intravenous immunoglobulin; PE, plasma exchange; SCIG, subcutaneous immunoglobulin; AHSCT, autologous hematopoietic stem cell transplantation; DPN, diabetic polyneuropathy; CI, confidence interval; NF-155, neurofascin-155; NF-140, neurofascin-140; NF-186, neurofascin-186; CNTN-1, contactin-1; CASPR1, contactin-associated protein 1; DSP, diabetic sensorimotor polyneuropathy; MMP-9, matrix metalloproteinase-9; CCM, corneal confocal microscopy; PDN, paraproteinemic demyelinating neuropathy; anti-MAG: anti-myelin-associated glycoprotein; DML, distal motor latency; CMAP, composite muscle action potential; ACR, American College of Rheumatology; POEMS, polyneuropathy, organomegaly, endocrinopathy, monoclonal gammopathy, and skin change; VEGF, vascular endothelial growth factor.
